# Renal Dysfunction and Recovery following Initial Treatment of Newly Diagnosed Multiple Myeloma

**DOI:** 10.1155/2018/4654717

**Published:** 2018-09-05

**Authors:** Benjamin A. Derman, Jochen Reiser, Sanjib Basu, Agne Paner

**Affiliations:** ^1^Section of Hematology/Oncology, University of Chicago, 5841 S. Maryland Ave, M/C 2115, Chicago, IL 60637, USA; ^2^Department of Medicine, Rush University Medical Center, Chicago, IL, USA; ^3^Division of Hematology/Oncology, Rush University Medical Center, Chicago, IL, USA

## Abstract

**Introduction:**

Renal insufficiency (RI) in Multiple Myeloma (MM) portends a higher tumor burden and worse prognosis. Reversal of RI in newly diagnosed MM (NDMM) improves patient outcomes, but it is unknown if there is a disparity in renal recovery in NDMM between African Americans (AA) and non-African Americans.

**Methods:**

A retrospective chart review was conducted of 690 patients with NDMM at Rush University Medical Center from 2005 to 2016. 118 patients (59 AA and 59 non-AA) with NDMM and an estimated glomerular filtration rate (eGFR) < 90 mL/min/1.73 m^2^ at the time of diagnosis were identified and analyzed. The time to best renal response and best eGFR achieved during initial myeloma therapy were tabulated.

**Results:**

Median eGFR at the time of diagnosis was similar between the AA and non-AA groups (47.89 versus 51.95, p=0.56). Median absolute change in eGFR after initial therapy was significantly higher in the AA (+33.64) versus the non-AA group (+21.07, p=0.00183). This difference remained whether the baseline eGFR at diagnosis was <90 or <60 mL/min/1.73 m^2^.

**Discussion:**

AA patients with NDMM treated in the era of novel agents have greater improvement in renal function in comparison to non-AA patients, regardless of myeloma response. The biological underpinnings for this disparity require further investigation.

## 1. Introduction

Renal insufficiency (RI) is present in roughly 20% of newly diagnosed multiple myeloma (NDMM) patients and over 50% of multiple myeloma (MM) patients will experience RI at some point during the course of their disease [1–3]. RI in multiple myeloma has been defined by the Internal Myeloma Working Group (IMWG) as a serum creatinine > 2 mg/dL or as an estimated glomerular filtration rate (eGFR) < 40 mL/min/1.73 m^2^ as calculated by the Modification of Diet in Renal Disease (MDRD) formula [4].

RI in MM portends a higher tumor burden and worse prognosis [5–8]. Survival appears to be tightly linked to the stage of chronic kidney disease (CKD), with survival decreasing in parallel with a decline in eGFR. In particular, those with an eGFR < 30 mL/min/1.73 m^2^ appear to have the worst prognosis [9]. Recent clinical trials have demonstrated that patients treated with “novel” agents, particularly proteasome inhibitors, are more likely to experience renal recovery. However, there is conflicting evidence as to whether reversal of RI in MM in the era of novel agents can improve overall survival. Of those studies that did show a survival difference, the prevailing theory is that reversal of RI in NDMM improves patient outcomes, but it remains inferior to patients whose renal function was normal at diagnosis [9–12].

The majority of patients in these trials were Caucasian, which may limit the external validity of the studies when considering a disease with a twofold predilection for African Americans (AA) and when addressing a population with a higher proportion of AA patients [13]. Moreover, AAs have a 5-times higher rate of stage 4 CKD and end stage renal disease (ESRD) in the United States compared to Caucasians. The cause for this disparity is multifactorial: less access to healthcare, higher incidence of causal diseases such as diabetes and hypertension, and differences in genetic factors (*APOL1* gene variants in AA populations) [14–17]. Recent insights suggest that APOL1 risk for kidney disease depends on the plasma levels of soluble urokinase receptor, an immune derived signaling molecule whose level is associated with lifestyle, infections, and even certain types of cancers [18].

Monitoring renal response has been standardized by IMWG's consensus statement on RI in MM (see Table 1). The eGFR, as calculated by the MDRD equation, can be used as a suitable substitute for creatinine clearance [4, 5]. The more recent CKD-EPI (Chronic Kidney Disease Epidemiology Collaboration) equation has been shown to be more accurate for estimating eGFR in the range of 60 to 90 mL/min/1.73 m^2^; however, the initial validation set was limited by lesser numbers of the elderly and of racial minorities [19].

Given the dearth of evidence regarding renal recovery in AAs receiving therapy for NDMM, the goal of this study is to compare renal recovery between AA and non-AA patients following initial treatment for NDMM.

## 2. Materials and Methods

A retrospective chart review was performed of patients with NDMM at Rush University Medical Center from January 1, 2005, to August 1, 2016. 690 charts were selected and reviewed through a myeloma registry; patients who were on hemodialysis for alternative reasons prior to diagnosis, had an eGFR > 90 mL/min/1.73 m^2^, or for whom records were incomplete were excluded via a thorough chart audit. The eGFR was calculated using the MDRD equation and confirmed using the CKD-EPI equation to ensure accurate eGFR assessment in the 60-90 mL/min/1.73 m^2^ range. 118 patients with NDMM and an eGFR < 90 mL/min/1.73 m^2^ (corresponding to National Kidney Foundation's chronic kidney disease stage 2 or worse) at the time of diagnosis were identified. Time to best renal response and the best eGFR achieved during initial myeloma therapy were recorded. MM response was recorded using the updated 2016 IMWG consensus criteria [20]. Continuous variables were compared between the two groups using the Mann-Whitney U test, and binary variables were compared using Fisher's exact test. The design of this study was approved by the hospital Institutional Review Board and is compliant with the Helsinki Declaration.

## 3. Results

### 3.1. Baseline Characteristics

A total of 118 patient records were reviewed, with 59 AA and 59 non-AA individuals with RI at the time of NDMM. The baseline patient characteristics at the time of diagnosis of multiple myeloma can be seen in Table 2. Both groups were comparable by age, gender, baseline eGFR, revised International Staging System for myeloma (R-ISS) and by anti-myeloma therapies received. The AA patient group presented with a higher incidence of hypertension, a greater degree of anemia, and a larger M-protein on serum protein electrophoresis compared to the non-AA group. There was a nonsignificant difference in the quantity of proteinuria at the time of diagnosis; proteinuria data was only available in 24 of the AA group and in 26 of the non-AA group. Cytogenetics by fluorescence in situ hybridization were evaluated from bone marrow biopsy samples in all patients. There was no significant difference in the cytogenetic risk as determined from the IMWG consensus on risk stratification [21]. In the AA group, there were 12 patients classified as having adverse risk cytogenetics compared to 14 patients in the non-AA group (p=0.825).

### 3.2. Renal Function at Diagnosis and after Recovery

Although median eGFR at the time of diagnosis of MM was similar between the AA and non-AA groups (47.89 versus 51.95 mL/min/1.73 m^2^, p=0.56), the median absolute change in eGFR after initial therapy was significantly higher in the AA group (+33.64 mL/min/1.73 m^2^) versus the non-AA group (+21.07 mL/min/1.73 m^2^, p=0.00183). This difference remained whether the baseline eGFR at diagnosis was <90 or <60 mL/min/1.73 m^2^ (Table 3). There was no significant difference in the median time to best renal response between the two groups (91 days in the AA group versus 79 days in the non-AA group, p=0.383). When substituting the CKD-EPI equation for the MDRD equation, 4 patients in the non-AA were reclassified as having a GFR > 90 mL/min/1.73 m^2^ and 0 patients in the AA group were reclassified. This did not have an appreciable effect on any of the eGFR variables.

### 3.3. Myeloma Response

The majority of patients were treated with a bortezomib-based regimen (86.4% for the AA group and 84.7% for the non-AA group, p=1). MM response rates to induction therapy were similar: very good partial response (VGPR) or better was achieved in 44.1% of AA and 35.6% of non-AA (p=0.452). There was not a significant difference in the percentage decrease in the involved-to-uninvolved serum free light chain ratio between the groups (87.39% in the AA group versus 92.88% in the non-AA group,* p*=0.103). 45.8% of AA individuals underwent autologous stem cell transplant (ASCT) compared to 64.4% of non-AA (p=0.0637). 80% of AA and 88% of non-AA patients received bisphosphonates (p=0.317, see Table 4).

## 4. Discussion

This is the first study to analyze disparities in renal dysfunction and recovery between AA and non-AA individuals with newly diagnosed multiple myeloma (NDMM). We demonstrate that, in our institution, AA patients with NDMM treated in the era of novel agents have greater improvement in renal function in comparison to non-AA patients, irrespective of myeloma response.

Prior studies examining renal recovery during treatment for NDMM have shown a positive correlation with overall survival; however, they have had limited external validity as they have primarily investigated Caucasian subjects. Our present work raises the question of whether there may be some biologic underpinning that accounts for the difference in renal recovery between AAs and non-AAs.

It is important to note that the IMWG diagnostic criteria for MM and the consensus statement on RI in MM are limited as they pertain to renal function in MM. Renal insufficiency in MM is defined as a creatinine clearance <40 mL/minute or serum creatinine >2 mg/dL. However, this cutoff is far below what is required to make the diagnosis of chronic kidney disease (CKD). This represents a key missed opportunity: early identification (and possibly treatment) of patients with renal insufficiency and MM. We argue that it is both reasonable and prudent to include patients with an eGFR <90 mL/min/1.73 m^2^, corresponding to patients with stage 2 CKD or worse. We have included a subanalysis of patients with a GFR <60 mL/min/1.73 m^2^ as well, corresponding to patients with stage 3 CKD or worse. In that same vein, we have eschewed the use of the IMWG renal response criteria and used the absolute change in eGFR from baseline in order to best quantify the renal response in our patients. The weaknesses of both the renal insufficiency criteria for MM and the renal response criteria must be readdressed in future guideline statements to more accurately assess renal response in MM.

This single-institution retrospective study is limited by its lack of power to investigate the effect of renal recovery on overall survival amongst the two groups and whether there is an association between myeloma response and renal response. Few renal biopsies were performed on patients in this data set, which limits the ability to attribute MM as the root cause of renal disease. Though serial serum free light chain measurements were performed reliably, the same cannot be said for serial proteinuria assessments which were missing. Delineating acute kidney injury from CKD in the setting of MM has historically been a challenge, owing to the overlapping contributions to renal injury by light chain cast nephropathy, volume depletion, radiologic contrast media, hypercalcemia, and non-steroidal anti-inflammatory agents used for bone pain prior to diagnosis [22]. It is possible that changes in muscle mass or dietary intake could have accounted for changes in the calculated eGFR over time. The median time to achieving best eGFR in this study (79-102 days) makes this less likely to have had an effect. Serum cystatin-C may be superior to creatinine in evaluating early renal dysfunction, and could be considered for future studies [23]. Moreover, nearly all MM patients undergo several lines of therapy during the course of their disease; our study only investigates renal recovery after the initial therapy modality.

## 5. Conclusion

Given that renal recovery in NDMM is known to impact overall survival, our findings suggest that further studies should be done to elucidate the differences in the epidemiology and disease biology that could account for the racial disparities in renal dysfunction and recovery. A promising explanation may lie in the interplay between* APOL1* gene expression and circulating soluble urokinase plasminogen activator receptor (suPAR) [24]. The* APOL1* G1 and G2 gene variants, which are prevalent in individuals with recent African ancestry and absent in Caucasians, are known risk factors for developing CKD and progression to ESRD in AAs [25]. Furthermore, it has been shown recently that APOL1-related decline in renal function is dependent on circulating suPAR levels, [24] which itself has been implicated in the onset and progression of CKD [26]. A murine model has been identified with “bone marrow immature myeloid cells (Sca-1^lo^Gr-1^lo^) as cellular sources of suPAR”; however, the human correlate has not yet been determined [27]. Myeloid lineage cells make up the bone marrow tumor microenvironment in MM and have clearly been shown to “promote [MM] cell survival, proliferation, and chemoresistance.” [28] Based on this evidence and the data that we present here, we speculate that the myeloid cells in the MM microenvironment may cause suPAR levels to rise and that antimyeloma therapies may act by altering this environment and lead to a resultant decrease in suPAR levels. This would provide an additional pathway for renal dysfunction and recovery in MM and might explain the racial differences in renal recovery described here. This presents an exciting potential mechanism that requires further investigation. In summary, our study suggests that AA patients with MM and renal disease experience greater recovery in kidney function with initial therapy. The biology underlying this interesting finding requires further study.

## Figures and Tables

**Table 1:** Renal response criteria*∗*.

**Renal Response**	**Baseline eGFR (mL/min/1.73m** ^**2**^ **)**	**Best CrCl Response**
Complete Response	< 50	≥ 60 mL/min

Partial Response	< 15	30-59 mL/min

Minor Response	< 15	15-39 mL/min
	15-29	30-59 mL/min

*∗*Adapted from the IMWG consensus statement on renal insufficiency in newly diagnosed multiple myeloma.

**Table 2:** Baseline data and patient characteristics.

	**AA (n=59)**	**Non-AA (n=59)**	***p*-value**
**Age (median)**	67.21	64.4	*p=0.372*

**Gender**			

Male	23	32	*p=0.140*

Female	36	27	

**Comorbidities**			

Hypertension	46	31	*p=0.0064*

Diabetes Mellitus	18	11	*p=0.1991*

Human Immunodeficiency Virus	1	0	*p=1*

Hepatitis C Virus	1	0	*p=1*

Systemic Lupus Erythematosus	1	1	*p=1*

Congestive Heart Failure	10	8	*p=0.799*

Chronic Kidney Disease	9	6	*p=0.582*

**Laboratory Data (median)**			

Hemoglobin (g/dL)	9	10.6	*p<0.001*

Platelets (10^9^/L)	194	206	*p=0.126*

eGFR (MDRD, mL/min/1.73 m^2^)	47.89	51.95	*p=0.522*

**Myeloma Parameters (median)**			

Protein Gap (g/dL)	5.9	4.15	*p=0.00241*

Lactate Dehydrogenase (U/L)	216	182.5	*p=0.400*

Beta2-Microglobulin (mg/L)	5.15	4.98	*p=0.742*

Urine Protein (mg/24 hrs)	279.5	1218	*p=0.192*

Serum Free Light Chain Ratio (Involved/Uninvolved)	70.37	164.96	*p=0.103*

M-protein (g/dL)	3.2	2	*p=0.0139*

% Bone Marrow Plasmacytosis	50	40	*p=0.053*

Light Chain only	12	13	*p=0.841*

Adverse Risk Cytogenetics*∗*	12	14	*P=0.825*

**Concurrent Amyloid**	4	2	*p=0.679*

**R-ISS Stage**			

1	4	10	*p=0.153*

2	35	39	*p=0.568*

3	20	10	*p=0.056*

**Criteria for Treatment**			

Hypercalcemia (Calcium > 11 mg/dL)	13	13	*p=1*

eGFR <60 mL/min/1.73 m^2^	45	37	*p=0.161*
eGFR 60-90 mL/min/1.73 m^2^	14	22	

Anemia (Hemoglobin < 10 g/dL)	32	17	*p=0.0086*

Bone disease	31	29	*p=0.854*

**Therapy Received**			

Triplet	24	27	*p=0.71*

Doublet	31	31	*p=1*

Other	4	1	*p=0.364*

**Bortezomib-based**	50	50	*p=1*
Bortezomib/Dexamethasone	24	23	*p=0.884*
Bortezomib/Lenalidomide/Dexamethasone	12	17	*p=0.353*
Cyclophosphamide/Bortezomib/Dexamethasone	11	6	*p=0.225*
Other	3	4	*p=0.705*

Bisphosphonate	47	52	*p=0.317*

*∗*Includes deletion 17p, t(4;14), t(14;20), t(14;16), and/or 1q21 gain. Triplet = 3-drug combination consisting of a corticosteroid and 2 other antimyeloma therapies. Doublet = 2-drug combination consisting of a corticosteroid and another antimyeloma agent.

**Table 3:** Renal response following initial therapy for newly diagnosed multiple myeloma.

**For eGFR <90 mL/min/1.73 m** ^**2**^ ** (MDRD)**	**AA (n=59)**	**Non-AA (n=59)**	***p*-value**
eGFR at diagnosis (median)	47.89	51.95	*p=0.56*

Change in eGFR (median)	33.64	21.07	*p=0.00183*

Time to best eGFR (median days)	91	79	*p=0.383*

**For eGFR < 60 mL/min/1.73 m** ^**2**^ ** (MDRD)**	**AA (n=45)**	**Non-AA (n=37)**	

eGFR at diagnosis (median)	34.09	31.29	*p=0.597*

Change in eGFR (median)	35.64	21.83	*p=0.0278*

Time to best eGFR (median days)	97.5	102	*p=0.983*

**Required HD**	6	6	*p=1*

**Table 4:** Multiple myeloma response following initial therapy.

**Myeloma Response**	**AA (n=59)**	**Non-AA (n=59)**	***p*-value**
Complete Response	11	8	*p=0.617*

Very Good Partial Response	15	13	*p=0.829*

Partial Response	27	28	*p=1*

Minimal Response	6	6	*p=1*

Stable Disease	0	4	*p=0.119*

**Light Chain Response (median)**			

% Decrease in Involved/Uninvolved Serum Free Light Chain Ratio	87.39	92.88	*p=0.187*

**Proceeded to ASCT**	27	38	*p=0.0637*

ASCT = autologous stem cell transplant.
